# Animal Life

**Published:** 1863-07

**Authors:** 


Animal Life.—The French correspondent of *The Times* gives an account of some experiments recently made in Paris as to the most humane mode of depriving oxen and other animals of life. He says that "the conservator of the Paris slaughterhouses, being of opinion that the mode of slaughtering oxen by knocking them on the head with a heavy metal instrument must cause the animal excessive pain, endeavored to discover another mode to avoid this suffering, and at the same time to preserve the slaughtermen from the danger to which they are exposed in the performance of this disagreeable duty. He thought that enervation would accomplish this object, and this opinion was founded on the doctrine taught by physiologists, who assert that the separation of the spinal marrow at once destroys animal life. Experiments were tried on more than one hundred oxen, and it was demonstrated that although the ox was more quickly put to death, his sufferings were not the less excruciating, inasmuch as his entire vitality was preserved, and death did not ensue until after an agony of fifteen or sixteen minutes. These experiments were repeated on calves and sheep, and, in place of merely cutting the spinal marrow, the head was separated from the body, in order to observe the degree of vitality which would still remain in each of the separated parts. A calf was suspended, and a butcher's boy cut his head off with a knife. The operation was accomplished in a quarter of a minute. The head was immediately placed on a table, and it lost two ounces and a half of blood in the space of six minutes. During the first minute all the muscles of the face and neck were agitated with rapid convulsions, and during the two following minutes the convulsions assumed another character. The tongue was stretched out of the mouth, which opened and closed alternately; the nostrils opened as if the animal experienced a difficulty of breathing. The convulsions became more active when the tongue or nostrils were pricked with a needle. When the hand was applied to the mouth or nostrils, respiration was felt to be continued by the air entering and coming out. When a finger was brought within an inch of the eye, in the direction of the pupil, the eye was quickly closed, as if it wished to avoid the touch of the finger, and the same result followed at several intervals. At length the eye did not close until the eyelid was touched. It was remarked that the eye remained closed as long as the finger remained in contact with it. These phenomena became gradually weaker, and ceased entirely after four minutes. Even then, when the spinal marrow was pricked with a needle, the convulsions recommenced in the entire face, tongue and eyes. After the sixth minute all contraction ceased. While these experiments were being performed, the body, which remained suspended, was greatly agitated. The agitation ceased gradually, and was replaced by feeble contractions, which continued more than an hour. But this was always observed, in whatever manner the throat was cut. Forty calves and fifty sheep were decapitated, and they all presented the same phenomena. The director of the Paris slaughterhouses convinced himself by these experiments that an ox suffered more by being decapitated than by being struck down with a heavy bar of iron; and, that the bar, by producing an immediate stupefaction, prevents the animal from suffering, while the bleeding, immediately effected, deprives him of life before the head recovers sensation."

It is obvious that in this record of the experiments, the painless nature of reflex movements is not properly considered and the experimenter should procure the assistance of a good physiologist in order to explain the character of the results observed. In that case he will probably arrive at very different conclusions from those above stated.

